# Association between anticholinergic burden and dementia in UK Biobank

**DOI:** 10.1093/geroni/igab046.3361

**Published:** 2021-12-17

**Authors:** Jure Mur, Simon Cox, Riccardo Marioni, Tom Russ, Graciela Muniz-Terrera

**Affiliations:** University of Edinburgh, Edinburgh, Scotland, United Kingdom

## Abstract

Previous studies on the association between the long-term use of anticholinergic drugs and dementia report heterogenous results. This variability could be due to, among other factors, different anticholinergic scales used, and differential effects of distinct classes of anticholinergic drugs. Here, we use 171,775 participants of UK Biobank with linked GP prescription records to calculate the cumulative annual anticholinergic burden (ACB) and ascertain dementia diagnoses through GP- and inpatient records. We then use Cox proportional hazards models to compare 13 anticholinergic scales and anticholinergic burden (ACB) due to different classes of drugs in their association with dementia. We find dementia to be more strongly predicted by ACB than by polypharmacy across most anticholinergic scales (standardised ORs range: 1.027-1.125). Furthermore, not only the baseline ACB, but the slope of the longitudinal trajectory of ACB (HR=1.094; 95% CI: 1.068-1.119) is predictive of dementia. However, the association between ACB and dementia holds only for some classes of drugs – especially antidepressants, antiepileptics, and high-ceiling antidiuretics. Moreover, we do not find a clear relationship between reported anticholinergic potency and dementia risk. The heterogeneity in findings on the association between ACB and dementia may in part be due to different effects for different classes of drugs. Future studies should establish such differences in more detail and further examine the practicality of using a general measure of anticholinergic potency as it relates to the risk of dementia.

